# V-J combinations of T-cell receptor predict responses to erythropoietin in end-stage renal disease patients

**DOI:** 10.1186/s12929-017-0349-5

**Published:** 2017-07-11

**Authors:** Henry Sung-Ching Wong, Che-Mai Chang, Chih-Chin Kao, Yu-Wen Hsu, Xiao Liu, Wen-Chang Chang, Mai-Szu Wu, Wei-Chiao Chang

**Affiliations:** 1 Department of Clinical Pharmacy, School of Pharmacy, Taipei Medical University, No. 250, Wuxing St, Xinyi District, Taipei City, 11031 Taiwan; 2 Master’s Program for Clinical Pharmacogenomics and Pharmacoproteomics, School of Pharmacy, Taipei Medical University, No. 250, Wuxing St, Xinyi District, Taipei City, 11031 Taiwan; 3 Ph.D. Program for Biotechnology in Medicine, College of Medical Science and Technology, Taipei Medical University, No. 250, Wuxing St, Xinyi District, Taipei City, 11031 Taiwan; 4 Division of Nephrology, Department of Internal Medicine, Shuang Ho Hospital, No. 291, Zhongzheng Road, Zhonghe District, New Taipei City, 235 Taiwan; 5 Graduate Institute of Clinical Medicine, College of Medicine, Taipei Medical University, No. 250, Wuxing St, Xinyi District, Taipei City, 11031 Taiwan; 6 The Ph.D. Program for Translational Medicine, College of Medical Science and Technology, Taipei Medical University and Academia Sinica, No. 250, Wuxing St, Xinyi District, Taipei City, 11031 Taiwan; 7 Section of Hematology/Oncology, Department of Medicine, The University of Chicago, 5801 S Ellis Ave, Chicago, IL 60637 USA; 8 Graduate Institute of Medical Sciences, College of Medicine, Taipei Medical University, No. 250, Wuxing St, Xinyi District, Taipei City, 11031 Taiwan; 9 Department of Internal Medicine, School of Medicine, Taipei Medical University, No. 250, Wuxing St, Xinyi District, Taipei City, 11031 Taiwan; 10 Cancer Center, Kaohsiung Medical University Hospital, No. 100, Ziyou 1st Road, Sanmin District, Kaohsiung City, 807 Taiwan; 11 Department of Pharmacy, Wan Fang Hospital, Taipei Medical University, No. 111, Section 3, Xinglong Road, Wenshan District, Taipei City, 116 Taiwan; 12 Center for Biomarkers and Biotech Drugs, Kaohsiung Medical University, No. 100, Shiquan 1st Road, Sanmin District, Kaohsiung City, 807 Taiwan

**Keywords:** End-stage renal disease, Erythropoietin treatment, T-cell receptor repertoire, High-throughput sequencing

## Abstract

**Background:**

Anemia is common among end-stage renal disease (ESRD) patients who undergone hemodialysis. The total reduction of red blood cell (RBC) count is associated with poor prognosis in these patients. Although erythropoietin (EPO) has been used as an effective treatment for ESRD patients with anemia, a large number of patients still present poor responses to EPO treatment.

**Methods:**

We measured T-cell receptor sequencing profiles, including length of complementarity-deteremining region 3 (CDR3), intra- and inter-group (EPO resistant vs. responsive) clonotype diversity, V(D)J usage profiles and V-J combinations from ESRD patients and to investigate the correlation between these features and EPO treatment efficacy.

**Results:**

Our results revealed statistical significance in the top 3 ~ 15 most abundant joint distributions of Vβ/Jβ among the two groups, suggesting the importance of V or J gene utilization in the EPO response of ESRD patients.

**Conclusions:**

In summary, we provided evidence addressing the potential correlation between the immune repertoire and EPO response in ESRD patients.

**Trial registration:**

TMU-JIRB 201309026. Registered 16 October 2013.

**Electronic supplementary material:**

The online version of this article (doi:10.1186/s12929-017-0349-5) contains supplementary material, which is available to authorized users.

## Background

End-stage renal disease (ESRD), a final-stage kidney disease, is an important health issue worldwide. Increasing ESRD populations have become a substantial burden on global healthcare expenditures [1]. In addition, the rapid decline in renal function, defined as the glomerular filtration rate (GFR), is associated with an increased risk of cardiovascular and all-cause mortality [2]. According to KDIGO 2012 guidelines, the progression from chronic kidney disease (CKD) to ESRD is characterized by grade 5 CKD requiring renal replacement therapy such as kidney transplantation or hemodialysis. The United States Renal Data System (USRDS) Annual Data Report of 2014 indicated that Mexico (466.5 per million population), Taiwan (449.7 per million population), and the United States (358.7 per million population) are with the highest incidence rate of ESRD in 2012. Besides, Taiwan (2902.1 per million population), Japan (2365.2 per million population), and the United States (1975.5 per million population) are with the highest ESRD prevalence in 2012. In particular in Taiwan, a CKD epidemic area, around 60,000 ~ 70,000 ESRD patients receive dialysis each year, and consume 5.89% of the National Health Insurance budget [3].

The impairment of the renal endocrine role in ESRD patients can lead to anemia [4], and the incidence of secondary anemia increases as the GFR declines. In a healthy condition, renal secretion of erythropoietin (EPO) plays a crucial role in red blood cell (RBC) proliferation and differentiation, and insufficient EPO production in ESRD patients leads to both decreasing RBC production and a 30% ~ 70% shorter RBC lifespan [5]. In 2013, Lin *et al*. reported a significant decrease in the hemoglobin (Hb) level in CKD patients compared to non-CKD samples in both elderly (≥60 years old) and non-elderly (>60 years old) subjects using a large Taiwanese cohort (*n* = 3352). As anemia may lead to a significant increase of cardiovascular disease risk and faster renal function decline, managing anemia is therefore crucial for the quality of life of ESRD patients. As a good management of anemia will improve the clinical outcomes of ESRD patients, erythropoiesis-stimulating agents (ESAs) are therefore widely used in ESRD-associated anemia. However, some ESRD patients show poor responsibility to EPO treatment. The resistance to EPO was reported to be associated with adverse prognoses and poorer clinical outcomes [6, 7]. Patients with ESRD have increased levels of several cytokines that negatively regulate erythropoiesis, e.g. interleukin (IL)-1, IL-6, tumor necrosis factor (TNF)-α, and interferon [IFN]-ɣ [8, 9]. The potential mechanisms include the direct inhibition to the growth of erythroid precursor, induction of apoptosis, and disrupting iron metabolism. Besides, it is well known that ESRD patients possess disruption of the T-cell-mediated immune responses [10]. Expansion of the memory T-cell population, impairment of T-cell functions, and diminished regulatory T-cells were observed in patients with renal failure [11, 12]. Zal *et al*. indicated that clonal expansion of the CD4^+^/CD28^−^ T-cell subpopulation may contribute to poorer cardiovascular outcomes in ESRD patients who receive hemodialysis [13]. Although the strong associations between the T-cell-mediated immune reactions and ESRD have been characterized, the potential mechanisms that influence T cell activation, cytokines production and EPO responses in ESRD patients remain unclear. Immune repertoire sequencing has recently emerged as a powerful method for profiling of the TCR repertoire [14]. This TCR sequencing method quantifying individual clonotypes by sequencing gene segments on variable (V), diversity (D) and joining (J) regions on the TCR, and further depicting the diversity of TCR repertoires. Using this approach, we hypothesized that TCR diversity and clonality among ESRD patients is the key to link the aberrant cytokine production and eventually contribute to the EPO responses.

## Methods

### Subject recruitment

Peripheral blood mononuclear cells (PBMCs) of samples were collected after obtaining informed consent from subjects at Taipei Medical University Hospital. The samples were collected from patients with written informed consent. The experiment was approved by the institutional review board at the Taipei Medical University and the methods were carried out in accordance with relevant guidelines and regulations. According to the KDOQI Clinical Practice Guidelines, ESRD patients were diagnosed according to eGFR decline of <15 ml/min/1.73m^2^ over 3 or more months. ESRD patients often exhibit anemia, and recombinant human EPO therapy is required to recover the hemoglobin (Hb) levels to at least 11 g/dL. However, some patients have to use a higher dosage of EPO than others to achieve Hb level targets. In this study, we recruited seven ESRD patients, whose eGFR had declined by <15 ml/min/1.73m^2^ and who were under regular hemodialysis for 3 months or more, and had also used EPO treatment. Three patients with an erythropoietin resistance index (ERI) of >5 U/kg/week (range, 11.6 ~ 12.3 U/kg/week) were classified into an EPO-nonresponsive group, and 4 EPO responders were defined by an ERI of <5.0 U/kg/week (range, 0.84 ~ 4.81 U/kg/week). This criterion resulted in an ERI grouping the same as in previous studies [15–17].

### Complementary (c)DNA synthesis and a two-step polymerase chain reaction (PCR)

Whole blood was diluted 1:1 with phosphate-buffered saline (PBS), added gently to the top of Histopaque (Sigma), and centrifuged at 400 ×*g* for 30 min. The peripheral blood mononuclear cells (PBMCs) were harvested after centrifuging and washing with PBS. Sequentially, the RNeasy Mini Kit (Qiagen) and QIAshredder spin column (Qiagen) were applied to extract the RNA from PBMCs. A reverse-transcription (RT)-PCR was performed with an One-Step RT-PCR kit (Qiagen) plus iRepertoire Primers (iRepertoire) according to the manufacturer’s instructions. cDNA templates were amplified by performing a Multiplex PCR with a Multiplex PCR kit (Qiagen) and Illumina Communal Primers of the HTBI-M type (iRepertoire). After library preparation, reads were obtained using the Illumina MiSeq System (Illumina).

### Raw sequencing data preprocessing

Raw paired-end sequencing data were de-multiplexed by sample barcodes to the FASTA format. Then, sample indices were trimmed while removing the adapter (Additional file 1). The paired-end reads were further overlapped. Non-overlapping reads were discarded in a downstream analysis. When overlapping, the base with the higher phred score was taken, thereby improving the overall overlapping read quality. The merged sequencing data were filtered by the following criteria: reads with a length of <50 base pairs (bp); reads with any nucleotide with a phred quality score of <20; and reads with <50% nucleotide bases that met the criterion of a phred quality score of >20. Ultimately, this resulted in overlapping reads with ~350 bp, which was sufficient to cover the entire complementarity-determining region (CDR) 3. In addition, the read length distribution was visualized using the *seeFastqPlot* function in the *systemPipeR* package (Additional file 2).

### VDJ mapping and clonotype identification

The resulting FASTA files were subjected to CDR3β extraction, V, D, and J regions mapping, clonotype identification of PCR, and sequencing errors with using MiTCR software [18] with the following parameters: ‘species = hs’ and ‘gene = TRB’. A phred quality score of >25 was set as the threshold value for each base within the CDR3 region. In addition, we used the ‘eliminate these errors’ algorithm for the PCR and sequencing error correction, which corrects a maximal number of accumulated errors, while sacrificing 1% ~ 3% of the TCR β diversity, according to our experiments (data not shown). The mappability of each sample was calculated by the number of mapped reads divided by the total number of reads. After clonotype clusterization, clonotype enumeration was conducted by summing the count of reads that coded for the same clonotype sequence. The percentage of clonotypes was calculated by the number of clonotypes divided by the number of nucleotide clones; and the percentage of in-frame clonotypes was calculated by the number of in-frame clonotypes divided by the total number.

### TCR β repertoire data analysis

In-frame reads were used for all downstream analyses. The inverse Simpson’s index (1/D) was applied to identify a sample’s clonotype diversity with the *vegan* package. In addition, to avoid the influence of rare clonotypes, we also assessed the diversity of individual sample repertoires by calculating the proportion of the top 5 most-abundant clonotypes. This parameter is robust for sequencing depth, and therefore allows comparisons between individuals. We also evaluated *TRBV* and *TRBJ* gene usage to enable profiling of repertoire manifestations of EPO responsiveness in ESRD patients.

### Statistical analysis

R software and Bioconductor were applied for all downstream statistical tests and visualization tasks using in-house R scripts.

## Results

### Quality assessment of the TCR β repertoire sequencing data in ESRD patients

**Fig. 1:**
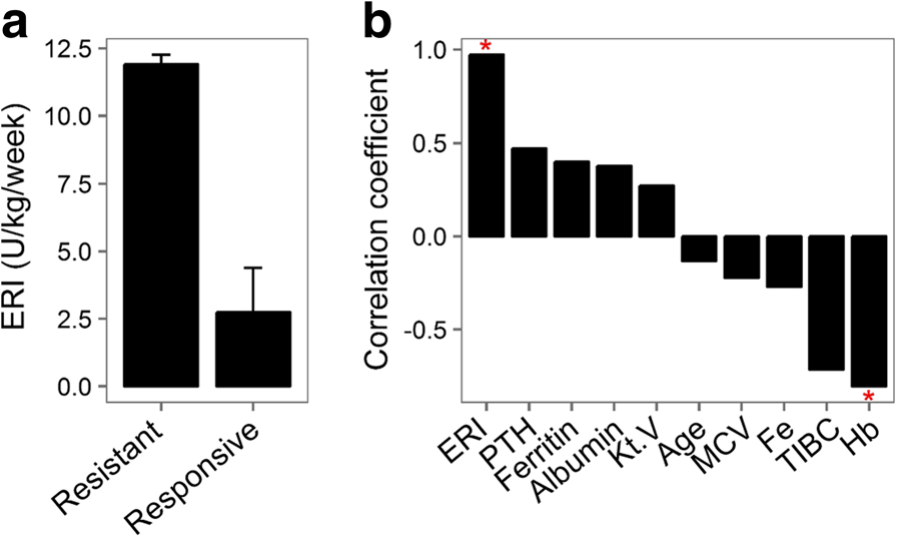
**a** The erythropoietin resistance index (ERI) levels in both erythropoietin (EPO) resistant and responsive groups (*p =* 0.017). **b** Correlation between end-stage renal disease patients’ clinical features and erythropoietin (EPO) responsiveness. Significant correlations between clinical features and EPO responsive status are denoted by “*”. Y-axis, mean square contingency coefficient

ESRD samples from seven patients were analyzed. Four of seven samples were defined as EPO-responsive (R) based on the erythropoietin resistance index (ERI) while the remaining three were defined as EPO-nonresponsive (NR) (Table 1). The ERI was calculated by the weekly weighted-adjusted EPO dose (U/kg/week) divided by the hemoglobin level (g/dL). The ERI of patients showed a significant difference between the EPO-responsive and EPO-nonresponsive groups (two-sample Fisher-Pitman permutation *p =* 0.017), with EPO responders and EPO non-responders having mean ERI values of 2.75 and 11.9, respectively (Fig. 1a). An association analysis of ESRD patients’ clinical features also revealed a significant correlation between the hemoglobin level and EPO responsiveness (two-sample Fisher-Pitman permutation *p =* 0.048, Table 2 and Fig. 1b).

**Table 1:** Patient characteristics of end-stage renal disease (ESRD) samples

Sample	Condition^a^	Gender^b^	Age (yr)	Comorbidities^c^
R1	Responder	M	65	DM + HTN + CAD
R2	Responder	F	43	CGN
R3	Responder	F	66	HTN + CGN
R4	Responder	M	62	DM + HTN
NR1	Non-responder (resistant)	M	63	DM + HTN
NR2	Non-responder (resistant)	F	46	CGN
NR3	Non-responder (resistant)	F	61	HTN + CGN

^a^ESRD patients’ erythropoietin (EPO) responsiveness. ^b^M, male; F, female. ^c^Diagnosed comorbidities: *DM* diabetes mellitus, *HTN* hypertension, *CAD* coronary artery disease, *CGN* chronic glomerulonephritis

**Table 2:** Correlation between end-stage renal disease patients’ clinical features and erythropoietin (EPO) responsiveness

	EPO responders	EPO non-responders	*P* value^a^
Age (yr)	59.0 ± 10.8	56.7 ± 9.3	0.746
Ferritin (mg/dL)	262.2 ± 117.3	430.7 ± 326.5	0.327
Fe (μg/dL)	71.8 ± 22.8	61.7 ± 17.9	0.507
TIBC (μg/dL)	258.0 ± 22.0	228 ± 3.6	0.080
Alb (g/dL)	4.2 ± 0.3	4.4 ± 0.1	0.355
MCV (fL)	92.8 ± 4.3	91.3 ± 2.1	0.584
PTH (pg/mL)	256.8 ± 102.1	455.3 ± 320.1	0.248
Kt/V	1.4 ± 0.3	1.6 ± 0.3	0.506
Hb (g/dL)	11.7 ± 0.9	9.9 ± 0.6	0.048^b^

^a^ *P* values were calculated by asymptotic two-sample Fisher-Pitman permutation test. *TIBC* total iron-binding capacity, *Alb* albumin, *MCV* mean corpuscular volumn, *PTH* parathyroid hormone, *Kt/V* (dialyzer clearance of urea)×(dialysis time)/(volume of distribution of urea); Hb, hemoglobin. ^b^ *P*-value < 0.05

To evaluate the analysis pipeline for the repertoire analysis, we exploited the 2 following parameters: (1) overlapping proportion and (2) mappability of reads. In the data-preprocessing step, individual TCR β cDNA sequences were subjected to overlapping. The robustness of overlapping step (Additional file 3) was found by a mean of 83.16% of overlap, and the small variation (standard deviation (SD) = 4.77%). In addition, after VDJ region determination, a mappability of 89.35% ± 4.91% further revealed the reliability of this approach (Additional file 4). The depths of CDR3 amino acid sequences (clonotypes) of individuals were also calculated. Only in-frame clone types, with nucleotide lengths that were divisible by 3, were subjected to all downstream analyses (Additional file 5).

### Repertoire diversity and EPO responsiveness in ESRD patients

**Fig. 2:**
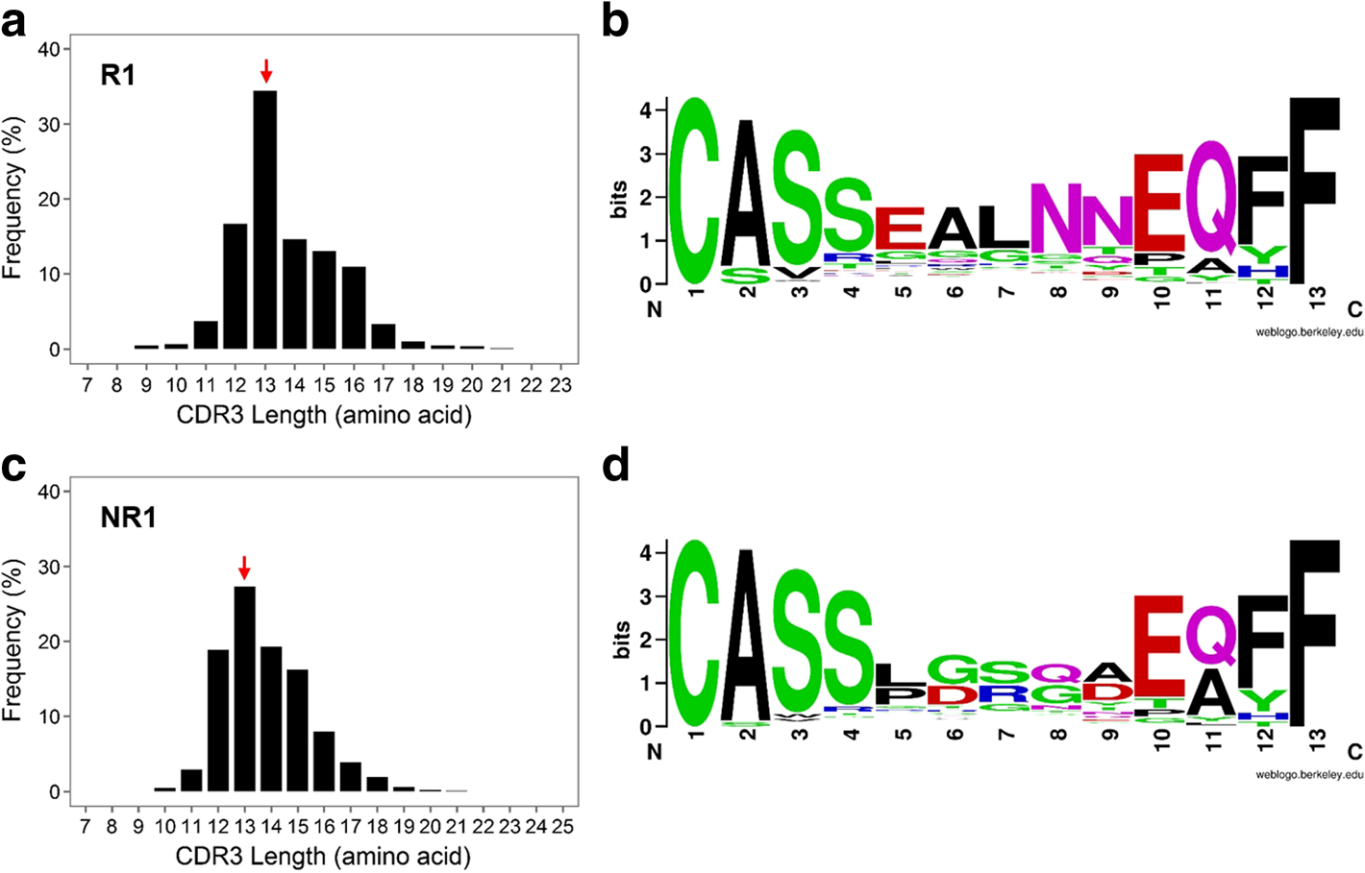
**a** Spectratype plot showing the distribution of T cell receptor (TCR) β complementarity determining region (CDR)3 length and their frequencies in sample R1. **b** 13-amino-acid CDR3 sequences of sample R1 were selected for WebLogo visualization. **c** Spectratype plot showing the distribution of TCR β CDR3 length and their frequencies in sample NR1. **d** 13-amino-acid CDR3 sequences of sample NR1 were selected for WebLogo visualization

To assess clonal expansions of TCR β in ESRD patients, we first depicted the β-CDR3 length distribution in seven ESRD patients (Fig. 2a, c and Additional file 6). As shown, substantial oligoclonal enrichment was observed in samples R1, R2, NR1, and NR3. The length of the most frequent β-CDR3 sequences ranging from 13 to 15 amino acids in these patients. In addition, the sequence pattern of the first 4 residues was very similar to PBMC of healthy individuals (Fig. 2b, d) [19].

We then determined the most frequent clonotype by percentage in both EPO responders and non-responders (Additional file 7a). We found that the most frequent clonotypes comprised 17.8%, 14.26%, 2.01%, 7.80%, 9.50%, 2.81% and 8.64% of reads in samples R1, R2, R3, R4, NR1, NR2, and NR3, respectively. In addition, we found 2, 2, 0, 1, 3, 0, and 2 clonotypes with frequencies of >5% in samples R1, R2, R3, R4, NR1, NR2, and NR3, respectively (Table 3).

**Table 3:** Most frequently emerging T-cell receptor (TCR) β sequences that constituted a proportion greater than 5% of all sequences

Sample*	CDR3 amino acid sequence	CDR3 length (nt)	Percentage	V gene	J gene	D gene
R1	CASSEALNNEQFF	39	17.76%	*TRBV2*	*TRBJ2–1*	*TRBD2*
	CAKGRVPYEQYF	36	6.83%	*TRBV2*	*TRBJ2–7*	*TRBD2*
R2	CACSASSSYEQYF	39	14.16%	*TRBV2*	*TRBJ2–7*	*TRBD2*
	CASSYSSQEQFF	36	5.33%	*TRBV6–3*	*TRBJ2–1*	*TRBD1*
R4	CATMGGYTF	27	7.71%	*TRBV6–5*	*TRBJ1–2*	*TRBD1*
NR1	CASSFSPHEQYF	36	9.43%	*TRBV28*	*TRBJ2–7*	*TRBD1*
	CASSLGSQAEAFF	39	8.21%	*TRBV7–8*	*TRBJ1–1*	*TRBD1*
	CASSPDRGDEQFF	39	6.23%	*TRBV12–3*	*TRBJ2–1*	*TRBD1*
NR3	CASSSGQDTEAFF	39	8.56%	*TRBV9*	*TRBJ1–1*	*TRBD1*
	CASSPQGAYEQYF	39	8.39%	*TRBV9*	*TRBJ2–7*	*TRBD1*

*R3 and NR2: no clonotype with a frequency > 5%

We further quantified the overall diversity of clonotypes using the inverse Simpson’s index (1/D), a mathematical index for assessing diversity for which the higher value means the greater the diversity. No significant difference in diversity indices between EPO responders and non-responders was observed (two-sample Fisher-Pitman permutation *p =* 0.642, Additional file 7b).

**Fig. 3:**
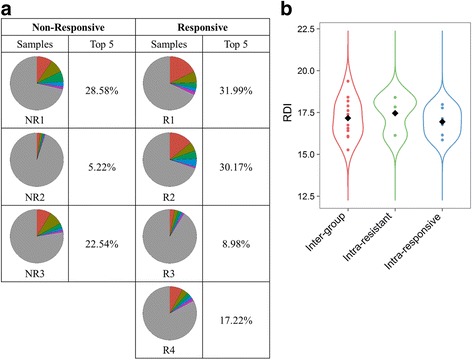
Clonal diversity of T cells in end-stage renal disease patients. **a** T-cell receptor (TCR) repertoires are depicted to show the individual diversity in erythropoietin (EPO) responders compared to EPO non-responders. Colors represent the abundance of each clone in percentages. **b** Comparison of pairwise repertoire diversity indexes (RDIs) of clonotype distribution across sample groups

To further explore the difference between the TCR β repertoires in EPO responders and non-responders in terms of the clone type pattern, the sum of proportions of the top 5 clones was employed to compare the diversity in each sample (Fig. 3a). The 5 most abundant TCR β clone types constructed 22.1% ± 10.9% of sequences in EPO responders, compared to 18.8% ± 12.1% in EPO nonresponders (two-sample Fisher-Pitman permutation *p =* 0.682). We further calculated the pairwise clonotype repertoire diversity index (RDI) [20] among inter-group and intra-group samples, however, no significant difference was found across different groups (Kruskal-Wallis rank sum *p =* 0.792, Fig. 3b). Taken together, we concluded that no significant correlation between TCR β diversity and EPO responsiveness was observed.

### Characterization of gene usage of *TRBV* and *TRBJ* in ESRD patients

**Fig. 4:**
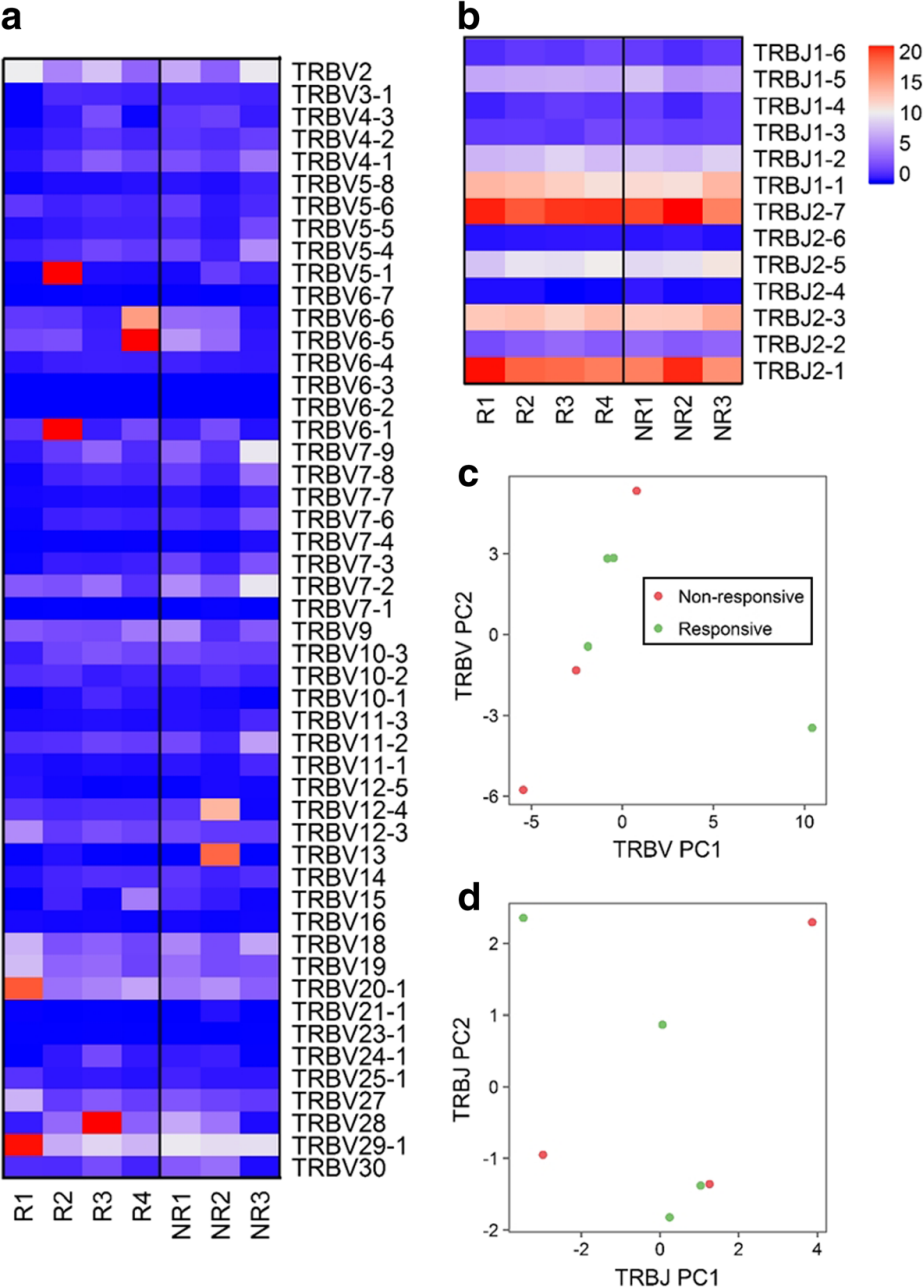
**a** Heatmap showing *TRBV* gene expression in all ESRD samples. **b** Heatmap showing *TRBJ* gene expression in all ESRD samples. **c** Figure depicting principal component analysis (PCA) result of *TRBV* gene usage across EPO responders and non-responders. **d** Figure depicting PCA result of *TRBJ* gene usage across EPO responders and non-responders

We next assess the gene usage of *TRBV* and *TRBJ* in EPO responders and non-responders. We observed that except several overexpression of specific *TRBV* genes, most ESRD patients shared a similar pattern of TRBV usage (Fig. 4a), regardless their responsiveness to EPO treatment. In addition, these patients also shared very similar *TRBJ* gene usage profiles (Fig. 4b). To validate this observation, we further conducted PCA analysis using *TRBV* and *TRBJ* gene usage frequencies. As result shown, EPO responders and non-responders were indistinguishable on the first and second principal component, suggesting the similar frequency of *TRBV* (Fig. 4c) and *TRBJ* (Fig. 4d) gene usage across two groups. Therefore, our results suggested that EPO responders and non-responders shared very similar *TRBV* and *TRBJ* gene usage profiles, despite some patient-specific gene enrichment pattern (especially on *TRBV* gene) was observed.

### Specific joint TRBV/TRBJ2–7 gene usage was enriched in EPO responders

**Fig. 5:**
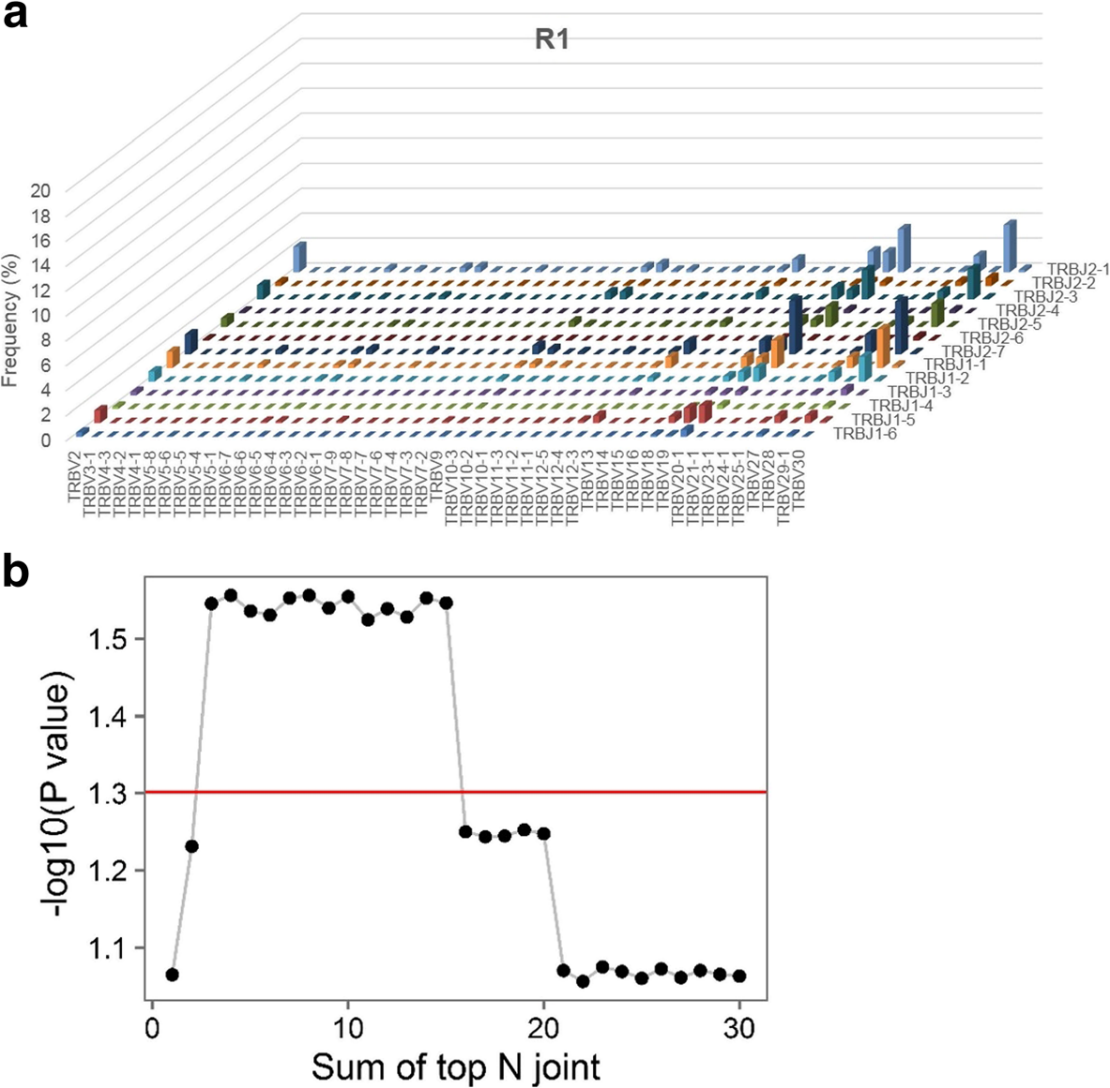
**a** 3D bar plots quantifying joint distributions of *TRBV* and *TRBJ* in R1 patients. The x-axis represent *TRBV* genes, and the z-axis represent *TRBJ* genes. Y-axis represents frequency of each joint Vβ/Jβ rearrangement. **b** Correlations between the sum of top 1 ~ 30 most abundant joint distributions of Vβ/Jβ to erythropoietin (EPO) responsiveness in ESRD patients

To clarify *TRBV* and *TRBJ* gene usage profiles in ESRD patients, we assessed the joint distribution between genes usages of Vβ and Jβ (Fig. 5a and Additional file 8). The top frequency of *TRBV*/*TRBJ* comprised 4.30%, 3.92%, 4.48%, 4.52%, 1.94%, 4.42% and 1.87% in samples R1, R2, R3, R4, NR1, NR2, and NR3, respectively. The high abundance (>3%) of joint Vβ/Jβ usage was observed in R1 (*TRBV20–1*/*TRBJ2–1, TRBV20–1*/*TRBJ2–7*, *TRBV29–1*/*TRBJ2–7, TRBV29–1*/*TRBJ1–1, TRBV29–1*/*TRBJ2–1, TRBV29–1*/*TRBJ2–7*), R2 (*TRBV5–1*/*TRBJ1–1, TRBV5–1*/*TRBJ2–1, TRBV5–1*/*TRBJ2–7*, *TRBV6–1*/*TRBJ2–1*, and *TRBV6–1*/*TRBJ2–7*), R3 (*TRBV28*/*TRBJ2–1, TRBV28*/*TRBJ2–7*), R4 (*TRBV6–5*/*TRBJ2–1, TRBV6–5*/*TRBJ2–7*), and NR2 (*TRBV13*/*TRBJ2–7*). However, we found no high abundance joint Vβ/Jβ usage in NR1 and NR3. In short, we observed high abundance rearrangements in all EPO responders (4/4, 100.0%) in contrast to 1 EPO non-responders (1/3, 33.3%; Chi-square *p =* 7.6 × 10^−9^).

**Fig. 6:**
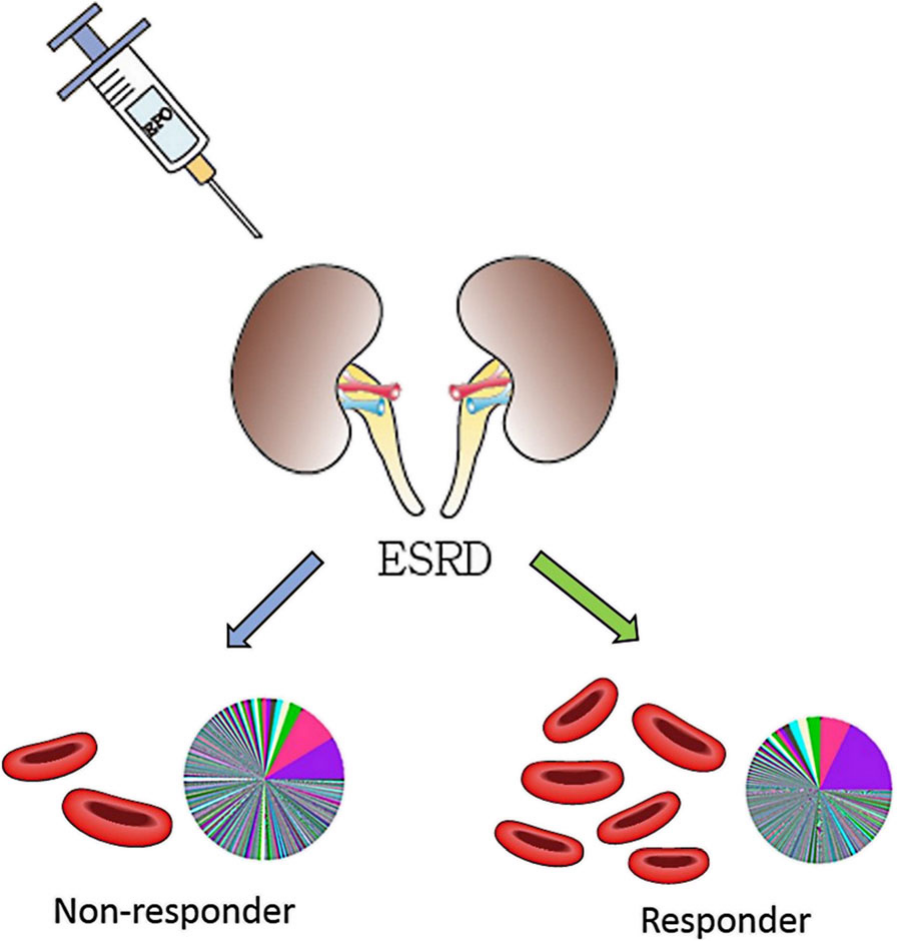
Schematic showing the TCR repertoire analysis focused on EPO responsiveness in ESRD patients. The *x* axis indicated the summation of top V-J combination(s) and the *y* axis indicated the significant value (−log_10_[*P* value]) regarding the sum of top V-J combination(s) across EPO responders and EPO nonresponders

To further assess the potential clinical utility of joint Vβ/Jβ profiles, we then asked whether joint Vβ/Jβ distributions may predict the EPO responsiveness of ESRD patients. We quantified the top abundant joint distributions of Vβ/Jβ, and this revealed a significant difference in the sum of top 3 to top 15 joint Vβ/Jβ distribution proportions between EPO-responsive and EPO-nonresponsive ESRD patients (two-sample Fisher-Pitman permutation *p* < 0.05, Fig. 5b). Together, we demonstrated different Vβ/Jβ usage patterns among EPO responders and non-responders, providing evidence for the correlation between TCR-mediated immune aberrations and EPO responsiveness (Fig. 6).

## Discussion

In this study, we identified high abundance rearrangements in all EPO responders compared to EPO non-responders, and showed that EPO responders had a higher sum of the top 3 to 15 sequence proportions than EPO non-responders.

The immune profiles of ESRD patients can be very complicated, as both suppression and activation of the immune system may coexist in patients [21]. Chronic immune system activation, which implies expansion of T-lymphocytes and elevated levels of pro-inflammatory cytokines (including interleukin [IL]-1, tumor necrosis factor [TNF]-α, and interferon [IFN]-ɣ) and anti-inflammatory cytokines (including IL-10 and IL-13), which were secreted by regulatory T cells or other immune suppressive cell populations, may contribute to the poor responsiveness to EPO [22]. These cytokines can affect the process of erythropoiesis and are associated with low EPO sensitivity. In the aspect of T-cell phenotypes, Cooper *et al*. conducted flow cytometry experiments and revealed significant CD28 loss of both CD4+ and CD8+ T cells in poor EPO responders, which implied that the T cells may lose the costimulatory factors for full activation [23]. But whether the first activation signal induced by T cell receptor was turned on is still unknown. Therefore, it is important to investigate the TCR constituents (especially clonality) that may contribute to beneficial EPO treatment responses via adaptive immune effects. Nest generation sequencing technique provided us a very good platform to study T-cell receptor profiles in ESRD patients, which were poorly reported before.

We separately demonstrated the results of TCR repertoire analyses in diversity and specificity aspects to depict the hallmarks of TCR-mediated adaptive immunity in ESRD patients who received EPO treatment. As shown, we found no significant difference in TCR β repertoire diversity in either EPO responders or non-responders. Diversity index that measures biodiversity in ecological systems has been adopted to quantify the TCR diversity [24, 25]. Since the elevated IL-10 was observed in EPO non-responders compared to the normal controls, we hypothesize that those enriched clones in our non-responder group such as NR1 and NR3 may indicate the clonal expansion of Treg population, although the further studies are needed to verify this. Meanwhile, we noted the individual difference of intra-group TCR β repertoire diversity in both patient groups, for which inverse Simpson’s diversity indexes ranged 25 ~ 420 in EPO responders and 47 ~ 641 in EPO non-responders, indicating distinct immune environments among individual ESRD patients.

In the current study, we clearly showed that partial expansion of T cells was observed in all EPO responders which might imply the initiation of adaptive immune response induced by TCR. However, whether those expanded T-cell clones also secreted cytotoxic cytokines and factors need to be examined in future studies. Due to the lack of the human leukocyte antigen (HLA) types information of patients, identified different common CDR3 sequences in ESRD patients may indicate the different antigens loaded in different HLA molecules among patients. Our pilot study will strongly inspire the following researches to correlate HLA, immune-phenotypes and cytokines together to understand the complicated immune environment in ESRD patients.

As shown in Fig. 2 we identified highly enriched Vβ/Jβ rearrangements in EPO responder group, which may represent that EPO responders have their own preference on V and J usages to generate the specific CDR3 regions for immune responses. In addition, although the enriched Vβ/Jβ rearrangement was also detected in NR2 who is in non-responder group, this patient has very diverse CDR3 clonal types. In other words, the enriched Vβ/Jβ rearrangement was not correlated with enriched CDR3 sequences in this non-responder. This interesting result implies that the mechanism of the complicated TCR rearrangement for immune responses may be quite different between EPO responder group and non-responder group.

We mentioned several limitations of our study in the following: Firstly, the sample size is relatively small to make a strong conclusion, and may limit the power of the study. However, we still observed the enrichment of particular V-J combinations in EPO-resistant in compared to EPO-responsive ESRD patients. Therefore, we may expect a more concrete conclusion regarding the observed correlations in larger sample cohort. In considering that there are so many factors that may confound the observed correlation of TCR profiles across two groups, we assessed the possible confounders and found no significant difference between these covariates across EPO responders and non-responders. However, the correlations between TCR clonotypes and immune-related factors (pro-inflammatory cytokines and anti-inflammatory cytokines) in ESRD patients remains to be elucidated. In addition, the human leukocyte antigen (HLA) gene profiles have not been assessed in our study, which may also limit the generalizability of the results to the whole population.

## Conclusions

In summary, we demonstrated a DNA sequencing-based method to characterize T-cell immunity in ESRD patients and measured the T-cell repertoire complexity to reveal similarities and differences between T-cell repertoire profiles underlying immune-pathological mechanisms of EPO responsiveness in ESRD patients (Fig. 6). As TCR repertoire behavior may affect the immune responses [26], our study provided a better understanding of the clonality of T-lymphocytes in ESRD patients that may explain the role of TCR-mediated adaptive immunity in EPO responsiveness.

## Additional files

Additional file 1: Schematic showing steps of T-cell receptor (TCR) β repertoire sequencing data processing. (DOCX 25 kb)

Additional file 2: Overlapping read length distribution plot of end-stage renal disease patients. QC, quality control. (DOCX 260 kb)

Additional file 3: Summary of repertoire sequencing output and overlapping proportions in end-stage renal disease samples. (DOCX 14 kb)

Additional file 4: Mappability to variable/diversity/joining (VDJ) regions in end-stage renal disease patients. (DOCX 13 kb)

Additional file 5: Summary of T-cell receptor (TCR) β clonotype statistics. (DOCX 14 kb)

Additional file 6: T-cell receptor (TCR) β complementarity determining region (CDR)3 size spectratype plots of amino acid clonotypes from end-stage renal disease patients. The CDR3 length is defined as the number of amino acids between the conserved positions CDR3, i.e., cysteine (Cys)104 and phenylalanine (Phe)118. The color of the bar is annotated by the number of corresponding nucleotide read counts in the samples. (DOCX 155 kb)

Additional file 7: Quantification of T-cell repertoire diversity in end-stage renal disease (ESRD) patients. a The clonotype distribution plots of ESRD patients. The x-axis and y-axis are log10 scaled. The value of 1/D represents the T-cell receptor (TCR) β repertoire diversity. b Comparison of TCR repertoire diversity (log-scaled) between erythropoietin (EPO) responders and EPO resistants (*p* = 0.642) (DOCX 232 kb)

Additional file 8: 3D bar plots quantifying joint distributions of TRBV and TRBJ in end-stage renal disease (ESRD) patients. (DOCX 517 kb)

## Abbreviations

Bp: Base-pair
CDR3: Complementarity-determining region 3
CKD: Chronic kidney disease
EPO: Erythropoietin
ERI: Erythropoietin resistance index
ESAs: Erythropoiesis-stimulating agents
ESRD: End-stage renal disease
GFR: Glomerular filtration rate
Hb: Hemoglobin
HLA: Human leukocyte antigen
HTS: High-throughput sequencing
IFN: Interferon
IL: Interleukin
PBMCs: Peripheral blood mononuclear cells
PBS: Phosphate-buffered saline
PCA: Principal component analysis
PCR: Polymerase chain reaction
RBC: Red blood cell
RDI: Repertoire diversity index
RT: Reverse-transcription
SD: Standard deviation
TCR: T-cell receptor
TNF: Tumor necrosis factor
USRDS: United States Renal Data System
