# Supplementary figures and images for: V-J combinations of T-cell receptor predict responses to erythropoietin in end-stage renal disease patients
Source: J Biomed Sci. 2017 Jul 11;24:43. doi: 10.1186/s12929-017-0349-5 (PMC5504791; doi:10.1186/s12929-017-0349-5)

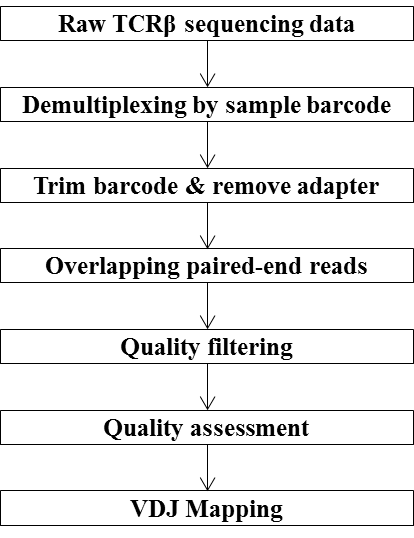

Supplement: Supplementary file 1 — Schematic showing steps of T-cell receptor (TCR) β repertoire sequencing data processing. (DOCX 25 kb) [file 12929_2017_349_MOESM1_ESM.docx]

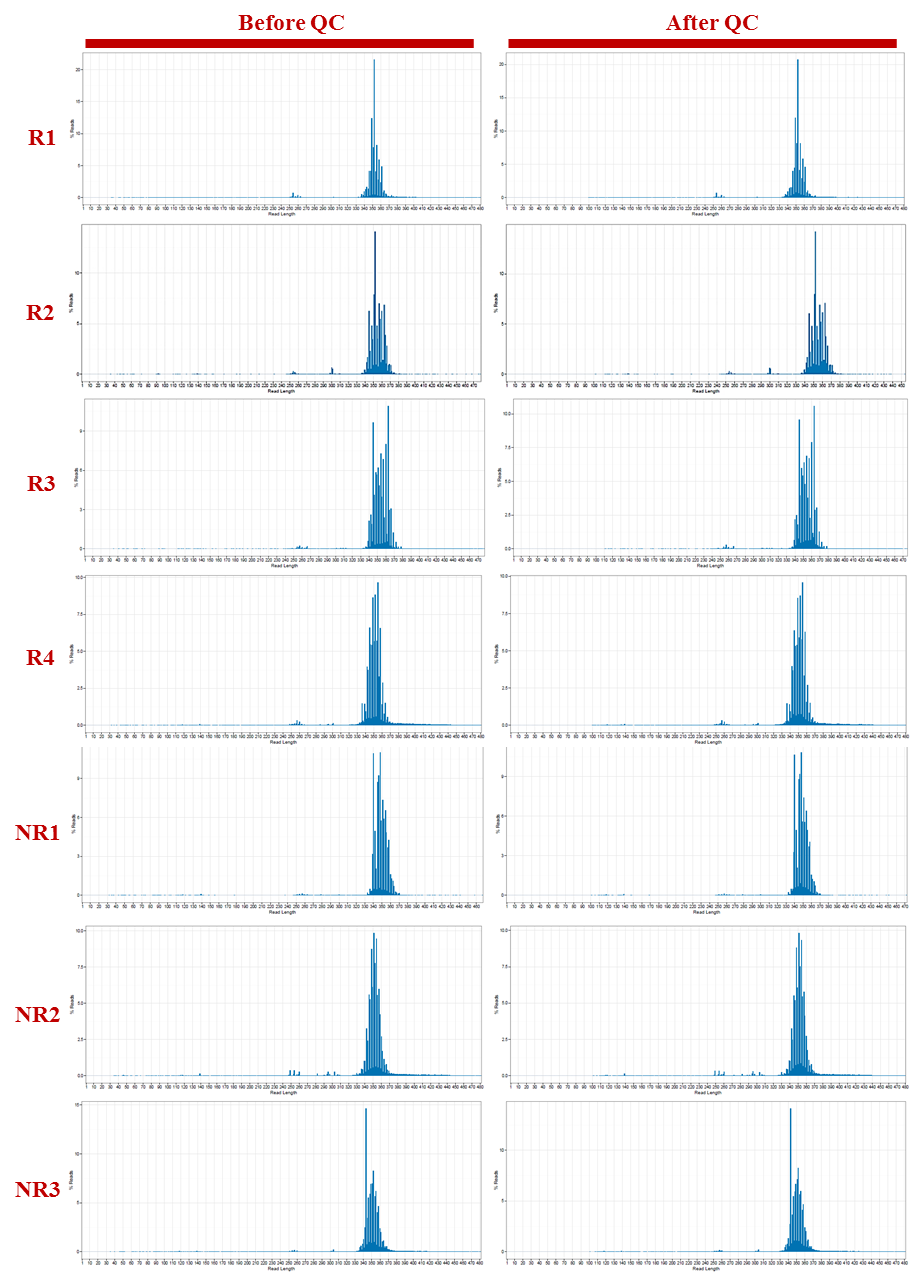

Supplement: Supplementary file 2 — Overlapping read length distribution plot of end-stage renal disease patients. QC, quality control. (DOCX 260 kb) [file 12929_2017_349_MOESM2_ESM.docx]

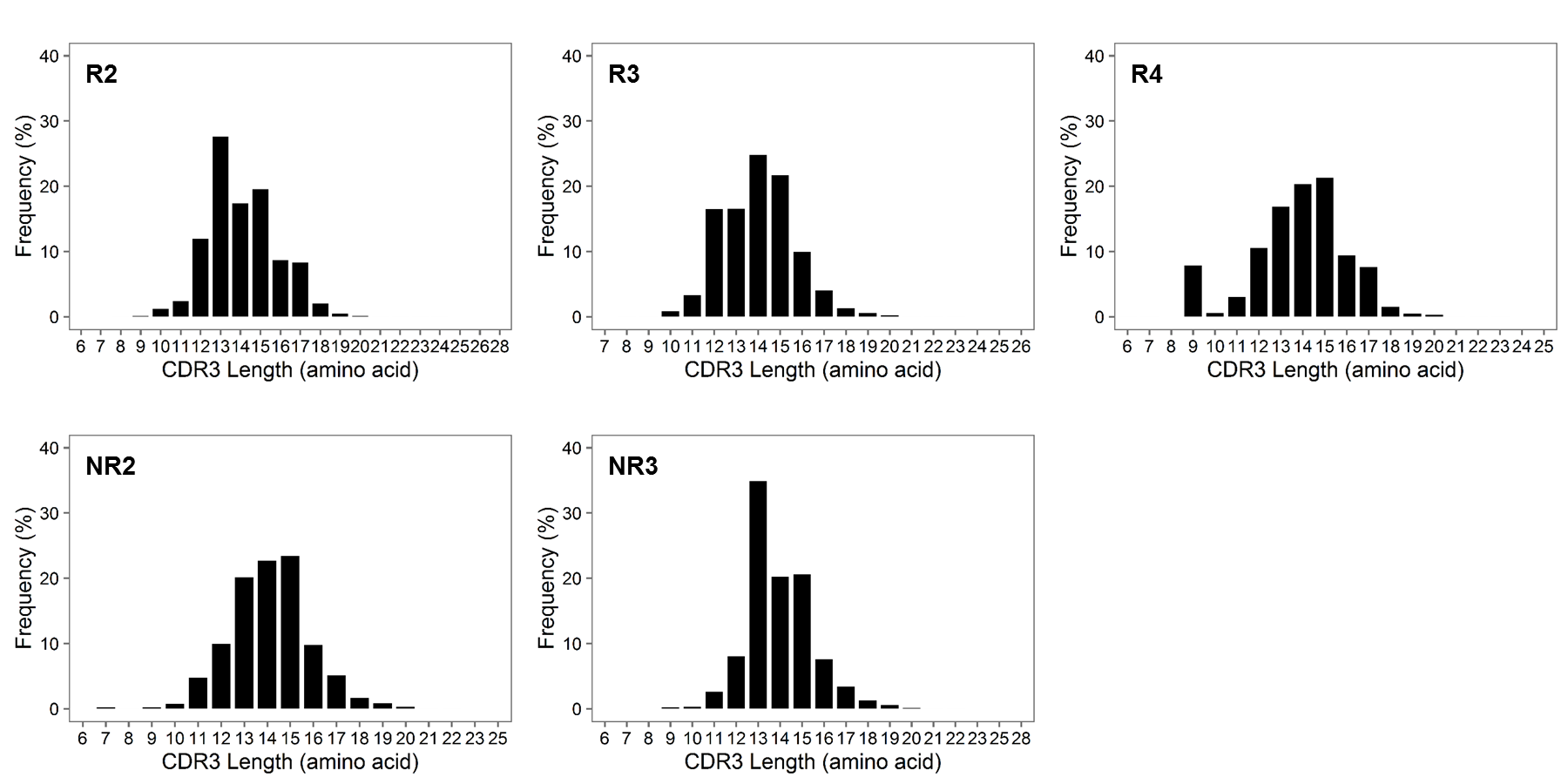

Supplement: Supplementary file 6 — T-cell receptor (TCR) β complementarity determining region (CDR)3 size spectratype plots of amino acid clonotypes from end-stage renal disease patients. The CDR3 length is defined as the number of amino acids between the conserved positions CDR3, i.e., cysteine (Cys)104 and phenylalanine (Phe)118. The color of the bar is annotated by the number of corresponding nucleotide read counts in the samples. (DOCX 155 kb) [file 12929_2017_349_MOESM6_ESM.docx]

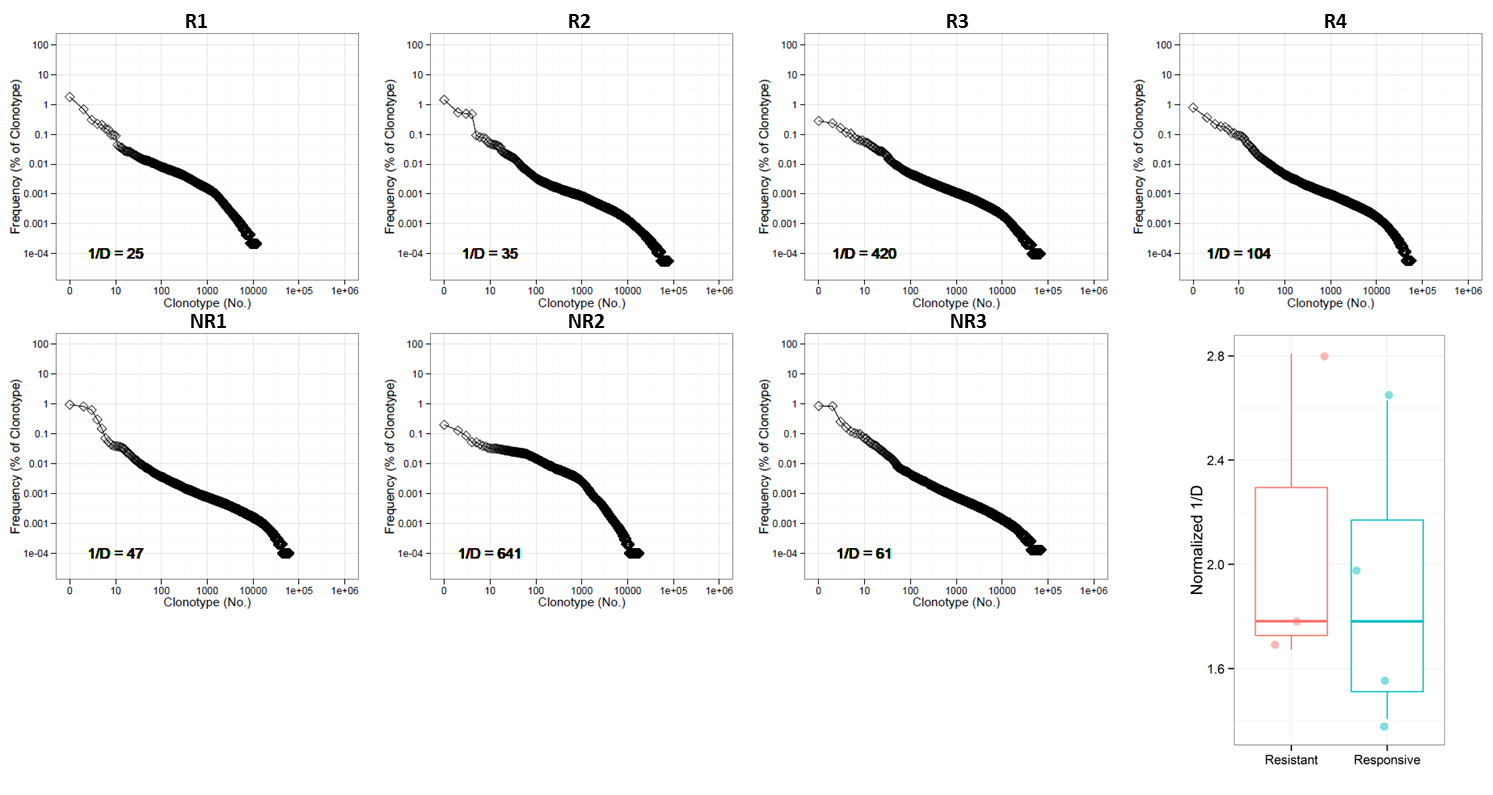

Supplement: Supplementary file 7 — Quantification of T-cell repertoire diversity in end-stage renal disease (ESRD) patients. a The clonotype distribution plots of ESRD patients. The x-axis and y-axis are log10 scaled. The value of 1/D represents the T-cell receptor (TCR) β repertoire diversity. b Comparison of TCR repertoire diversity (log-scaled) between erythropoietin (EPO) responders and EPO resistants (p = 0.642) (DOCX 232 kb) [file 12929_2017_349_MOESM7_ESM.docx]

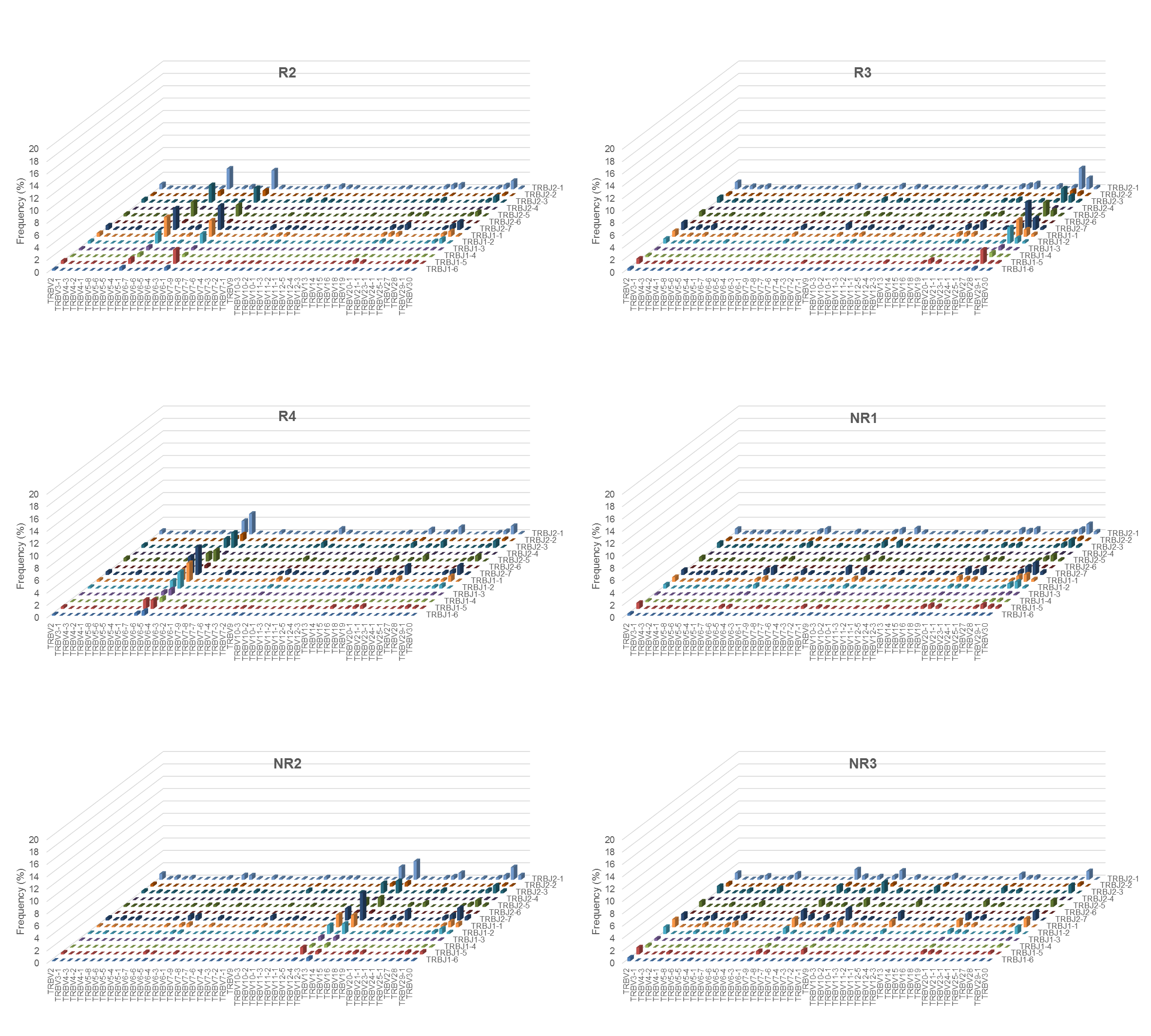

Supplement: Supplementary file 8 — 3D bar plots quantifying joint distributions of TRBV and TRBJ in end-stage renal disease (ESRD) patients. (DOCX 517 kb) [file 12929_2017_349_MOESM8_ESM.docx]
