# Supplementary material for: V-J combinations of T-cell receptor predict responses to erythropoietin in end-stage renal disease patients
Source: J Biomed Sci. 2017 Jul 11;24:43. doi: 10.1186/s12929-017-0349-5 (PMC5504791; doi:10.1186/s12929-017-0349-5)
Supplement: Supplementary file 3 — Summary of repertoire sequencing output and overlapping proportions in end-stage renal disease samples. (DOCX 14 kb) [file 12929_2017_349_MOESM3_ESM.docx]

| **Additional file 3** Summary of repertoire sequencing output and overlapping proportions in end-stage renal disease samples. | | | |
| --- | --- | --- | --- |
| Patients | Total reads ^a^ | Overlapping reads ^b^ | Percentage |
| R1 | 719,330 | 525,211 | 73.01% |
| R2 | 2,293,901 | 1,969,404 | 85.85% |
| R3 | 1,442,389 | 1,217,811 | 84.43% |
| R4 | 2,140,452 | 1,872,835 | 87.50% |
| NR1 | 1,230,055 | 1,046,188 | 85.05% |
| NR2 | 1,462,810 | 1,201,797 | 82.16% |
| NR3 | 1,081,455 | 910,074 | 84.15% |
| ^a^Read counts after demultiplexing. ^b^Read counts of overlapping paired-end reads. | | | |
