# Supplementary material for: V-J combinations of T-cell receptor predict responses to erythropoietin in end-stage renal disease patients
Source: J Biomed Sci. 2017 Jul 11;24:43. doi: 10.1186/s12929-017-0349-5 (PMC5504791; doi:10.1186/s12929-017-0349-5)
Supplement: Supplementary file 4 — Mappability to variable/diversity/joining (VDJ) regions in end-stage renal disease patients. (DOCX 13 kb) [file 12929_2017_349_MOESM4_ESM.docx]

| **Additional file 4** Mappability to variable/diversity/joining (VDJ) regions in end-stage renal disease patients. | | |
| --- | --- | --- |
| Sample | Number of mapped reads | Total mappability ^a^ |
| R1 | 460,141 | 87.68% |
| R2 | 1,790,075 | 90.99% |
| R3 | 1,016,259 | 84.67% |
| R4 | 1,775,135 | 94.81% |
| NR1 | 1,009,461 | 96.58% |
| NR2 | 1,022,762 | 84.04% |
| NR3 | 787,853 | 86.67% |
| ^a^Mappability (%) = (sum of mapped reads/total reads) × 100. | | |
