# Supplementary material for: V-J combinations of T-cell receptor predict responses to erythropoietin in end-stage renal disease patients
Source: J Biomed Sci. 2017 Jul 11;24:43. doi: 10.1186/s12929-017-0349-5 (PMC5504791; doi:10.1186/s12929-017-0349-5)
Supplement: Supplementary file 5 — Summary of T-cell receptor (TCR) β clonotype statistics. (DOCX 14 kb) [file 12929_2017_349_MOESM5_ESM.docx]

| **Additional file 5** Summary of T-cell receptor (TCR) β clonotype statistics. | | | | |
| --- | --- | --- | --- | --- |
| Sample | No. of clonotypes | Percentage of clonotypes | No. of in-frames | Percentage of in-frames |
| R1 | 12,416 | 97.90% | 12,291 | 96.92% |
| R2 | 81,269 | 95.92% | 82,021 | 96.81% |
| R3 | 69,785 | 96.61% | 70,183 | 97.16% |
| R4 | 59,555 | 97.29% | 58,578 | 95.70% |
| NR1 | 61,921 | 97.27% | 61,853 | 97.16% |
| NR2 | 18,759 | 97.59% | 18,324 | 95.33% |
| NR3 | 73,701 | 95.68% | 74,753 | 97.04% |
|  | | | | |
